# Streamlining Micronutrient Biomarker Statistical Analysis in Populations: An Introduction to the SAMBA R Package

**DOI:** 10.1016/j.tjnut.2023.06.024

**Published:** 2023-06-23

**Authors:** Hanqi Luo, Ty Beal, Tineka Blake, Madeleine Zeiler, Jiaxi Geng, E Rochelle Werner, O Yaw Addo, Parminder S. Suchdev, Melissa F. Young

**Affiliations:** 1 Hubert Department of Global Health, Rollins School of Public Health, Emory University, Atlanta, Georgia, United States; 2 Global Alliance for Improved Nutrition, Washington DC, United States; 3 University of Nottingham, Nottingham, United Kingdom; 4 Centers for Disease Control and Prevention, Atlanta, Georgia, United States

**Keywords:** micronutrients, biomarkers, statistical analysis, software, inflammation adjustment

## Abstract

Micronutrient deficiency is a common global health problem, and accurately assessing micronutrient biomarkers is crucial for planning and managing effective intervention programs. However, analyzing micronutrient data and applying appropriate cutoffs to define deficiencies can be challenging, particularly when considering the confounding effects of inflammation on certain micronutrient biomarkers. To address this challenge, we developed the Statistical Apparatus of Micronutrient Biomarker Analysis (SAMBA) R package, a new tool that increases ease and accessibility of population-based micronutrient biomarker analysis. The SAMBA package can analyze various micronutrient biomarkers to assess status of iron, vitamin A, zinc, and B vitamins; adjust for inflammation; account for complex survey design when appropriate; and produce reports of summary statistics and prevalence estimates of micronutrient deficiencies using recommended age-specific and sex-specific cutoffs. In this study, we aimed to provide a step-by-step procedure for how to use the SAMBA R package, including how to customize it for broader use, and made both the package and user manual publicly available on GitHub. SAMBA was validated by comparing results by analyzing 24 data sets on nonpregnant women of reproductive age from 23 countries and 30 data sets on preschool-aged children from 26 countries with those obtained by an independent analyst. SAMBA generated identical means, percentiles, and prevalence of micronutrient deficiencies to those calculated by the independent analyst. In conclusion, SAMBA simplifies and standardizes the process for deriving survey-weighted and inflammation-adjusted (when appropriate) estimates of the prevalence of micronutrient deficiencies, reducing the time from data cleaning to result generation. SAMBA is a valuable tool that facilitates the accurate and rapid analysis of population-based micronutrient biomarker data, which can inform public health research, programs, and policy across contexts.

## Introduction

Micronutrient deficiencies, also known as hidden hunger, are estimated to affect 1 in 2 preschool-aged children (PSC) and 2 in 3 nonpregnant women of reproductive age (WRA) [1]. Micronutrient deficiencies can lead to poor growth, intellectual impairment, and increased risk of morbidity and mortality [2]. Nutrition intervention programs, such as large-scale fortification, supplementation, and dietary diversification, can address this global burden. To effectively plan and implement these programs, a broad range of information is needed, such as data on food availability, food and nutrient intake, micronutrient status, and program coverage. Understanding the prevalence of micronutrient deficiencies in target populations is a critical aspect of nutrition research and measuring effect of nutrition programs [3].

The 2017 Global Nutrition Report proposed a “nutrition data revolution,” calling for “better use of data that is collected to create a more responsive information system” in policy decision making [3]. This call to action applies not only to low-income and middle-income countries but also to high-income countries where at least subpopulations are at increased risk for micronutrient deficiencies (such as iron deficiency in women). The Global Nutrition Report introduced a “nutrition data value chain” with 5 critical processes: data prioritization, creation and collection, curation, analysis, and interpretation and recommendation for decision making [4,5]. Much effort has been put into overcoming the early challenges in this chain [[6], [7], [8]]. For example, the recently updated Micronutrient Survey Manual and Toolkit provides comprehensive instructions for planning a national micronutrient survey [6], and the WHO guidelines on drawing blood, establishing best practices in phlebotomy, for obtaining high-quality blood samples in population surveys [9]. Although these guidelines provide recommendations on data analysis and presentation, little progress has been made in streamlining the statistical analysis of micronutrient biomarker data, which still requires advanced statistical skills (for example, expertise in analyzing surveys with complex design and incorporating the appropriate sampling frame), proficiency in computer coding, and identifying when to adjust for inflammation or apply age-specific and sex-specific cutoffs to define micronutrient deficiency [10]. Hence, organizations may need to hire senior statisticians specialized in micronutrient data or invest in building this capacity in their analysts. However, the time required to hire consultants or learn new techniques may result in delaying opportunities to make micronutrient biomarker data available during critical decision-making periods.

To improve use of micronutrient data, we developed the Statistical Apparatus for Micronutrient Biomarker Analysis (SAMBA) package in R software. This easy-to-use program can analyze biomarkers for iron, vitamin A, zinc, folate, and vitamin B-12 from 1 or multiple data sets simultaneously, adjust for inflammation and complex survey design when appropriate, and produce summary statistics of micronutrient deficiencies using recommended age-specific and sex-specific cutoffs. The objective of this article was to present the general structure and features of the SAMBA package. We aimed to explain the underlying theory behind the tool, provide the GitHub link (GitHub link: https://github.com/hanqiluo/SAMBA) for downloading the open-access package and the associated user manual, and give examples of potential applications of the tool. We also applied the SAMBA package to 24 data sets on WRA from 23 countries and 30 data sets on preschool-aged children from 26 countries and compared results with those generated by an independent analyst using R programming language. By following the detailed user manual and examples we provided, analysts can adapt the sample codes for analyzing their own micronutrient biomarker data.

### Challenges in Analyzing Micronutrient Biomarkers

The statistical analysis of micronutrient biomarker data poses challenges, such as harmonizing nonstandardized file formats, variable names, and units across surveys or countries in addition to the aforementioned challenges in adjusting for inflammation, selecting cutoff values, and applying statistical methods appropriate for limits of detection and the study design when applicable. These challenges are described in detail further.

### *File formats for micronutrient data sets*

Survey coordinators who design and implement surveys often do not analyze the collected data themselves, so analysts may receive data files in a format that is not compatible with the software they use. Although there are packages that can convert between different types of data files (for example, Stata, SAS, and Excel files), analysts still need to learn how to use these packages to convert the data format.

### *Variable names for micronutrient biomarkers*

Biomarkers can be stored using several variations in spelling and notation of variables names, which can create confusion for analysts. For example, the biomarker serum ferritin can be stored under the variable names serum_ferritin, serum.ferritin, sf, ferritin, or FER. This can be particularly challenging in the absence of a comprehensive codebook, which is always recommended but sometimes unavailable.

### *Micronutrient biomarker units*

Biomarkers can be expressed in either conventional units or the International System of Units [11]. Analysts may need to reference additional information such as molar mass of the biomarker and convert units before comparing the biomarker with an established cutoff value to define micronutrient deficiency.

### *Methods of inflammation adjustment*

Inflammation is known to affect many micronutrient biomarkers, which can lead to an overestimation or underestimation of the prevalence of deficiencies in a population [10,12]. To address the confounding effects of inflammation, several inflammation adjustment methods have been developed and used [13], such as changing micronutrient biomarker cutoffs (that is, using fixed higher or lower cutoff to define micronutrient deficiencies), exclusion (that is, removing observations with inflammation), correction factor approach (multiplying the observed micronutrient concentration by a fixed correction factor defined by individual inflammation status) [14], and Biomarkers Reflecting Inflammation and Nutritional Determinants of Anemia (BRINDA) inflammation adjustment method (that is, using a regression correction to adjust for the confounding effects of inflammation) [15]. We previously described the strengths and weaknesses of each approach and suggest using the BRINDA inflammation adjustment approach in both high and low inflammation settings because using a regression-correction approach may better consider the full range and severity of inflammation than would the exclusion or correction factor approaches that rely on dichotomous cutoffs to define inflammation [[15], [16], [17], [18]].

### *Cutoffs for micronutrient deficiencies*

To assess micronutrient deficiencies, measured values are compared with reference ranges or cutoff values established by recommendation of the manufacturers of biological assays, WHO guidance and guidelines documents, or by consensus of international experts. These cutoffs may be adjusted based on physiologic or environmental factors. For example, the serum zinc concentration cutoff used to determine zinc deficiency can be influenced by fasting status and time of data collection [19].

### *Limits of detections and imputation*

Limits for detections (LoDs) for micronutrient biomarkers refer to the lowest or the highest concentration of a micronutrient that can be reliably detected by an assay or measurement method [8]. LoDs are biomarker specific and subject to prelaboratory analytic, laboratory analytic, and postlaboratory analytic factors, such as source of blood collection, the specific assay used, the quality control mechanisms of the laboratory performing the analysis, and storage conditions for the assays [20]. It is recommended to thoroughly document source of blood collection, laboratory procedures, and assay details as standard reporting practice in national surveys and study trials. Values beyond the LoD may not be reliable; hence, imputation or other methods of data handling need to be implemented during the statistical analysis stage [21]. Although published methodologies exist for addressing LoDs specific to CRP, the task of developing similar approaches for LoDs associated with a wider array of micronutrient biomarkers needs further research.

### *Analytical approach for study design*

To accurately analyze data from a multistage complex survey design, analysts need to be familiar with sampling frames and the role that strata, clusters, and survey weights play in estimating descriptive statistics such as means, geometric means, and prevalence of deficiencies. It is important for researchers to provide well-documented information on sampling frames and for analysts to have a thorough understanding of these concepts to produce reliable and valid results from such studies.

### *Inconsistent and contradictory results*

Inconsistent and contradictory results from study replication by an independent analyst can compromise the validity of the findings [22]. This is particularly problematic when analytical methods and codes are not readily accessible, making it difficult to evaluate and diagnose flawed analyses. It is important for analysts to have a strong understanding of statistical methods and follow best practices for producing transparent and reproducible results.

### Introduction to the SAMBA Package

Although there are some statistical packages to address micronutrient biomarker analysis, such as the BRINDA SAS macro [23] and R package [24], they tend to focus on a specific component, such as BRINDA inflammation adjustment [10,15]. This means that analysts are required to use multiple packages and write their own codes to address the missing components. Therefore, an all-in-one statistical analysis software could efficiently address the aforementioned limitations. The SAMBA R package was developed as a comprehensive and user-friendly tool to estimate the prevalence of multiple micronutrient deficiencies for multiple data sets simultaneously. The SAMBA package also includes additional features, such as the ability to check input data sets for data-loading errors, implement analyses involving inflammation adjustment, support user-specified cutoff values for micronutrient deficiencies [8,15], and handle multistage complex survey design, where applicable. This section describes the algorithm of the SAMBA package, introduces a customized feature for analyzing additional micronutrient biomarkers, and presents the validation of the SAMBA package.

### Algorithm of SAMBA

**FIGURE 1:**
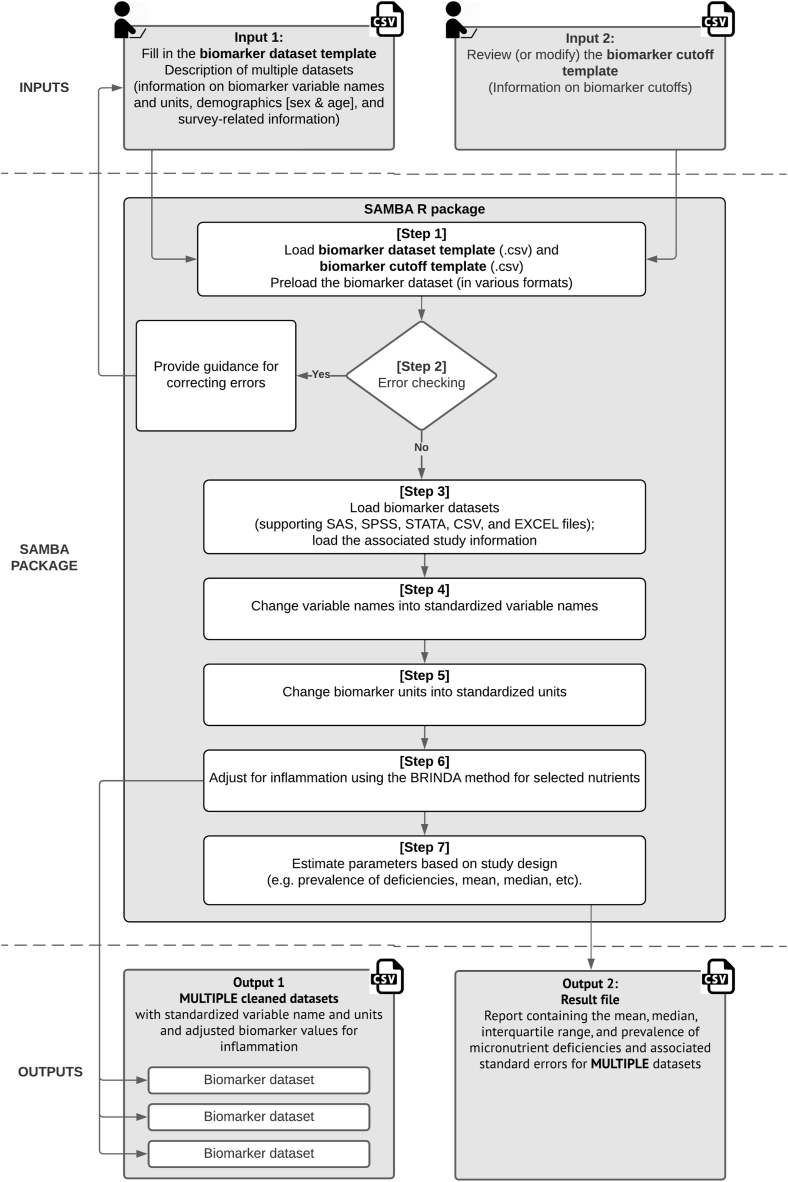
Overview of the SAMBA package inputs, core contents, and outputs. BRINDA, Biomarkers Reflecting Inflammation and Nutritional Determinants of Anemia; SAMBA, Statistical Apparatus of Micronutrient Biomarker Analysis.

The SAMBA package requires 2 input files: *1*) the biomarker data set template and *2*) the biomarker cutoff template. For the biomarker data set template, analysts need to specify the local directory used to store the data set in their computer, survey-related information (for example, strata, cluster, and survey weight variable names), demographic information (for example, age and sex variable names), and micronutrient biomarker variable names and units. In this template, analysts can include information for multiple data sets (1 column per data set). For the biomarker cutoff template, analysts need to review the existing cutoff values that vary by age and population group (for example, male, female, and pregnant women) and modify these values if desired. An overview of the SAMBA package inputs, core content, and outputs is shown in Figure 1.

The SAMBA package is easy to use, requiring only 5 lines of R programming code: users define the directories of the biomarker data set template and cutoff template in their computer, the number of biomarker data sets they want to analyze, and the directory and name for the output data sets. After all information is input, the SAMBA package will process micronutrient data using the following 7 steps:

Step 1 SAMBA will load the biomarker data set template and cutoff template and preload the biomarker data set(s), which can be in SAS, SPSS, Stata, CSV, or Excel.

Step 2 SAMBA will use the information in the biomarker data set template to check for errors in the input data (for example, if a user-specified variable exists in the data set) before starting analysis. If any detectable errors are found, SAMBA will provide guidance for correcting them.

Step 3 If no errors exist, the biomarker data sets will be officially loaded by SAMBA.

Step 4 SAMBA will standardize biomarker variables using the predefined names in the biomarker data set template.

Step 5 SAMBA will convert the units of the biomarkers to the International System of Units for further analysis.

Step 6 SAMBA will implement the BRINDA inflammation adjustment method on selected micronutrient biomarkers, such as serum ferritin, sTfR, RBP, serum retinol, and serum zinc (if applicable), using α(1)-acid glycoprotein (AGP) and/or CRP among PSC and WRA [18,25,26]. After inflammation adjustment, 1 cleaned data set per input data set will be exported in a CSV format. The cleaned CSV files contain standardized variable names, standardized units, and inflammation-adjusted biomarker values for all participants (output 1). If a data set neither contains AGP nor CRP or requires BRINDA inflammation adjustment for micronutrient biomarkers, SAMBA will bypass step 6, directly output cleaned data sets and proceed to step 7.

Step 7 SAMBA will estimate the summary statistics of micronutrient biomarkers such as the arithmetic mean, geometric mean, median, IQR, and prevalence of deficiencies with associated SEs and CIs for all input data sets. The output will be formatted as a CSV file suitable for use in reports or manuscripts (output 2).

These steps are executed automatically by the SAMBA package, making it easy for analysts to obtain reliable and valid results from their micronutrient data. To help analysts get started with the SAMBA package, we have provided detailed examples and a user manual on the GitHub page for the SAMBA package (https://github.com/hanqiluo/SAMBA). These examples provide step-by-step instructions on using the SAMBA package for a nutrition baseline survey in Kenya [27] and the US NHANES 2003–2006 [28]. These examples and the user manual will make it easy for analysts to adapt the sample code to their own micronutrient biomarker data.

### Customization and extension of the SAMBA package

The SAMBA package was designed to be generic, allowing analysts to use it to analyze a wide range of cutoffs and biomarkers beyond the default settings included in this package. Without any modification to the R package itself, the SAMBA package can be used to analyze any biomarkers with a binary outcome. For example, it can be used to estimate iron deficiency using total body iron (as opposed to the already built-in ferritin and sTfR analysis features in SAMBA) and other health conditions such as hypertension, hypoglycemia or hyperglycemia, and hypercholesterolemia. To include additional biomarkers in the analysis, analysts need to add the variable names, units, and survey weight variables (if applicable) to the existing biomarker data set template and the cutoff values for each additional biomarker to the existing cutoff template. Once the SAMBA package runs successfully, it will generate a clean data set (output 1) that includes individual participant’s information on the additional biomarker(s) and a summary result file (output 2) that contains descriptive statistics of the additional biomarker for the population. This allows analysts to easily and quickly expand the scope of their analysis to include a wider range of biomarkers without the need to modify the underlying R code.

The outputs of the SAMBA package (either the summary result file or clean data sets) can be connected with other nutrition information and health outcomes to address a range of public health research questions, such as examining the double burden of malnutrition, investigating the relation between micronutrient deficiencies and clinical diagnoses of maternal and child health, and measuring the effect of nutrition interventions in reducing micronutrient deficiencies.

### Validation of the SAMBA package

To validate the SAMBA package, we applied it to 30 PSC data sets from 26 countries and 24 WRA data sets from 23 countries from the BRINDA database (https://www.brinda-nutrition.org/brinda-countries). Using the SAMBA package, we estimated the prevalence of deficiencies; the arithmetic and geometric means; and the 25th, 50th, and 75th percentiles for a range of micronutrient biomarkers (such as serum ferritin, sTfR, RBP, serum retinol, serum zinc, serum folate, and serum B-12). Subsequently, an independent analyst used the same methods (for example, complex survey design, BRINDA inflammation adjustment, and use of identical cut offs) to obtain results using R programming software. We found that the results produced by SAMBA and the independent analyst were identical. Validation code can be found on GitHub (https://github.com/hanqiluo/SAMBA/tree/main/validation_code). This demonstrates the accuracy and reproducibility of the SAMBA package.

**FIGURE 2:**
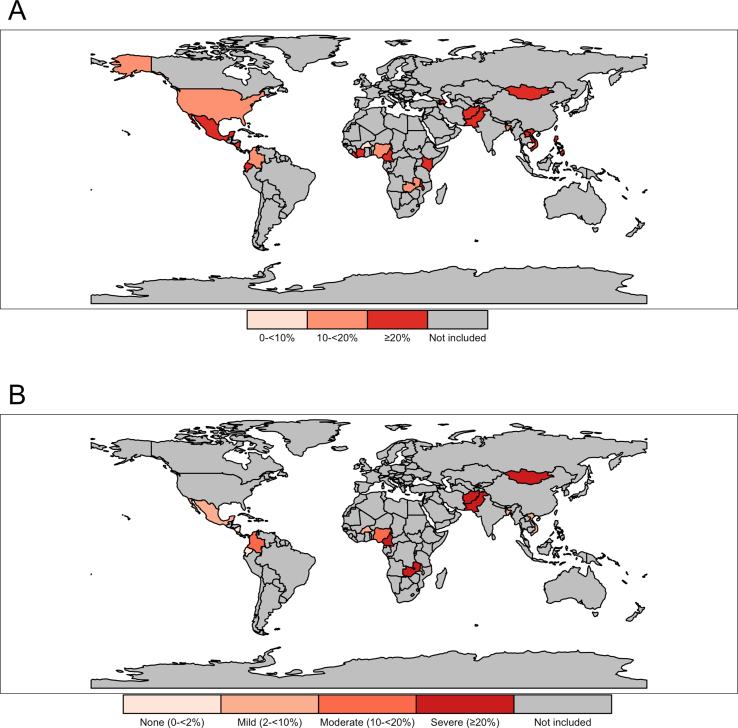
Prevalence of (A) iron deficiency and (B) vitamin A deficiency in preschool-aged children (age 6–59 mo) using BRINDA data sets from 26 countries. Iron deficiency defined as BRINDA inflammation-adjusted serum ferritin concentration of <12 μg/L. Vitamin A deficiency defined as BRINDA inflammation-adjusted serum retinol concentration of <0.7 μmol/L. BRINDA, Biomarkers Reflecting Inflammation and Nutritional Determinants of Anemia.

**FIGURE 3:**
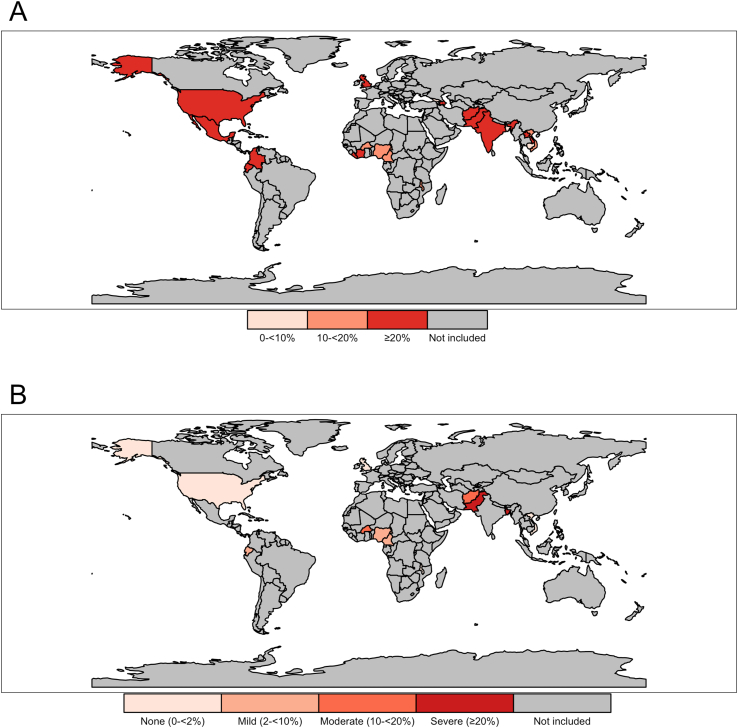
Prevalence of (A) iron deficiency and (B) vitamin A deficiency in nonpregnant women of reproductive age (15–49 y) using BRINDA data sets from 23 countries. Iron deficiency defined as BRINDA inflammation-adjusted serum ferritin concentration of <15 μg/L. Vitamin A deficiency defined as serum retinol concentration of <0.7 μmol/L. BRINDA, Biomarkers Reflecting Inflammation and Nutritional Determinants of Anemia.

As an example of the outputs that can be generated using SAMBA, we mapped the prevalence of iron deficiency using serum ferritin concentration adjusted for inflammation and the prevalence of vitamin A deficiency using serum retinol concentration adjusted for inflammation for PSC in Figure 2 and WRA in Figure 3. These maps show the spatial distribution of micronutrient deficiencies and can be used to inform public health policy and interventions.

## Discussion

### Summary

The SAMBA package provides a streamlined and efficient structure for the analysis of multiple micronutrient biomarkers for multiple data sets. Before the development of the SAMBA package, analysts needed to have a high level of statistical knowledge and proficiency with computer coding. The SAMBA package simplifies this process, making it accessible to novice analysts and allowing them to produce BRINDA inflammation-adjusted and survey-weighted estimates of micronutrient deficiencies more quickly and easily. This can help reduce the time from data cleaning to results and improve the efficiency of micronutrient data analysis.

### Advantages and application of SAMBA in micronutrient data analysis

Micronutrient deficiencies are an important aspect of malnutrition for countries of all incomes [29] and can exist in both underweight and overweight populations [30]. However, micronutrient deficiencies are frequently overlooked when assessing malnutrition owing to both the scarcity and limited utilization of data [29]. The Global Nutrition Report and other initiatives have emphasized the need to fill the data gap using a holistic approach that improves all components in the data value chain, from data prioritization, creation and collection, curation, and analysis to interpretation and recommendations [4,5]. The SAMBA package is a software tool that can fill the gap in the nutrition data value chain between data collection and interpretation for public health decision making. The SAMBA package reduces the time spent on *1*) learning the BRINDA adjustment method for inflammation, *2*) learning statistical skills (for example, analysis of complex survey design), and *3*) writing codes to analyze each biomarker separately. This makes it a valuable tool for improving the availability and use of micronutrient data in global public health decision-making and research settings.

The SAMBA package is particularly useful for countries that collect micronutrient data as part of their nutrition surveillance system and for organizations that monitor global micronutrient status. The ability of the SAMBA package to simultaneously analyze multiple micronutrient data sets can save time and effort and improve the efficiency of micronutrient data analysis. Another advantage of the SAMBA package is that it is built in R, a free and open-access software platform. This means that institutions and analysts do not need to invest in additional software to use the SAMBA package, which improves its accessibility. There is an increasing emphasis on building research capacity in public health nutrition in low-income and middle-income countries, and the SAMBA package can help to support these efforts by providing a user-friendly platform for analyzing micronutrient biomarker data. This can reduce the time and cost required for a micronutrient data analysis and facilitate the use of these data for local and international policy making. In addition, the SAMBA package allows analysts to easily extend their analyses to include additional biomarkers beyond the default biomarkers included in the package. This flexibility allows analysts to explore a wider range of micronutrient deficiencies, to link the outputs of the SAMBA package (either the summary result file or the cleaned data sets) with other nutrition information and health outcomes, and to better understand the associations between micronutrient deficiencies and noncommunicable diseases.

### Limitations of SAMBA

The SAMBA package also has several limitations. First, the SAMBA package can only produce accurate and unbiased results when the micronutrient biomarker data are of high quality and accurately reflect the population’s micronutrient status. SAMBA does not remove any outliers or values outside of the LoDs. For many surveys, such as NHANES, biomarker values are already cleaned and within a reasonable range. However, by default, the SAMBA package will recode zero values to 0.0001 to enable log transformation of biomarker values. To ensure the validity of micronutrient data results, besides rigorous training of laboratory technicians, we recommend analysts undertake several crucial preliminary steps before using the SAMBA package on their own data. This should include rigorous examination of the distribution of the micronutrient data to identify and manage outliers and implausible values, the scrutiny for duplicate entries, and the identification of missing values. Moreover, they should consider implementing suitable imputation techniques for handling missing data and values beyond LoDs [21]. These measures are essential to ensure the accuracy of the results generated through the SAMBA package. Second, SAMBA only provides inflammation-adjusted micronutrient values using the BRINDA inflammation adjustment method. Other inflammation adjustment methods, such as using correction factors [14] and the exclusion method [31], are not included in the SAMBA package. Third, the BRINDA inflammation adjustment method is only applicable to a limited set of nutrients (that is, serum ferritin, sTfR, RBP, serum retinol, RBC and serum folate, serum B-12, and serum zinc concentrations) in WRA and PSC, although SAMBA can be adapted to analyze all types of micronutrients among all population groups. To apply inflammation adjustment to other population groups, analysts are recommend to first examine the relation between nutrients and inflammation markers (AGP or CRP) and only apply the BRINDA adjustment algorithm if a statistical and biological relation exists [10]. Another important consideration is that serum zinc cutoff values can vary based on fasting status and the time of day [19]. Although SAMBA allows for variation in cutoff values based on population and age group, it does not consider fasting status or the time of a day. Hence, analysts are advised to subset the data set based on these factors, modify the cutoff template accordingly, and analyze SAMBA separately to ensure accurate results.

### Future directions of SAMBA

The SAMBA package has specific technical requirements. Analysts need to have R software installed and be equipped with basic R programming skills, which requires time and access to appropriate training materials. To facilitate use of the current SAMBA package version, we have designed training materials and a thorough, publicly available user manual and build the capacity of researchers and policy analysts (training materials are available on request). Plans to expand the current SAMBA tool include the addition of new features, such as updating SAMBA based on the latest development of new international consensus on micronutrient biomarker cutoff values and the BRINDA methods for inflammation adjustment. To ensure ease of use and widespread adoption, support materials such as workshops, online tutorials, and instructional YouTube videos will be developed and added to the BRINDA website (www.brinda-nutrition.org). In the longer term, we envision the development of a web-based tool based on the SAMBA package to further increase the accessibility of the method and decrease the time and resources required to make results of micronutrient biomarker data available.

## Conclusion

The SAMBA package simplifies the estimation of the prevalence of micronutrient deficiencies, which may reduce analysis errors and the time from data cleaning to presentation of results. SAMBA may facilitate the analysis of micronutrient biomarker data to support evidence-based nutrition research, programs, and policymaking.

## Author contributions

All authors contributed to the model design and interpretation of the data; HL: developed the method and wrote the article; T Beal: proposed the idea and assisted in method development and editing; T Blake, JG: assisted the code editing; OYA: provided statistical support; MZ, ERW: assisted with manuscript editing and provided useful insights; PS, MY: provided overall guidance on the project and participated in the manuscript editing; and all authors: critically revised the manuscript and approved the final version.

## Conflict of interest

The authors report no conflicts of interest.

## Funding

Supported by the US Agency for International Development Advancing Nutrition, the US Agency for International Development, Centers for Disease Control and Prevention, Eunice Kennedy Shriver National Institute of Child Health and Human Development, and HarvestPlus. This work was supported, in whole or in part, by the Bill & Melinda Gates Foundation INV-010744 and INV-002855. Under the grant conditions of the Foundation, a Creative Commons Attribution 4.0 Generic License has already been assigned to the Author Accepted Manuscript version that might arise from this submission.

## Disclaimer

The findings and conclusions in this report are those of the authors and do not necessarily represent the official position of the Centers for Disease Control and Prevention.
